# Supplementary material for: Advancing anesthesiology trainee proficiency in airway management via simulation-based training: a non-hypoxic apnea duration approach
Source: PeerJ. 2025 Jun 4;13:e19555. doi: 10.7717/peerj.19555 (PMC12145085; doi:10.7717/peerj.19555)
Supplement: Supplemental Information 2 [file peerj-13-19555-s002.docx]

**CONSORT 2010 Checklist of Information for a Randomized Trial**

| **Section/Topic** | **Item No.** | **Reported on Page** | **Checklist Item** |
| --- | --- | --- | --- |
| **Title and Abstract** |  |  |  |
| Title and abstract | 1a | Title Page | Identification as a randomized trial in the title |
|  | 1b | Page 1 (Abstract) | Structured summary of trial design, methods, results, and conclusions |
| **Introduction** |  |  |  |
| Background and objectives | 2a | Page 3-4 | Scientific background and explanation of rationale |
|  | 2b | Page 4 | Specific objectives or hypotheses |
| **Methods** |  |  |  |
| Trial design | 3a | Page 5 | Description of trial design (parallel, factorial) etc. |
|  | 3b | Page 5 | Important changes to methods after trial commencement |
| Participants | 4a | Page 5-6 | Eligibility criteria for participants |
|  | 4b | Page 5 | Settings and locations where the data were collected |
| Interventions | 5 | Page 6-7 | Precise details of interventions intended for each group |
| Outcomes | 6a | Page 7 | Completely defined pre-specified primary and secondary outcome measures |
|  | 6b | Page 7 | Any changes to trial outcomes after the trial commenced |
| Sample size | 7a | Page 8 | How sample size was determined |
|  | 7b | N/A | When applicable, explanation of any interim analyses and stopping guidelines |
| Randomization |  |  |  |
| Sequence generation | 8a | Page 5 | Method used to generate the random allocation sequence |
| Allocation concealment | 8b | Page 5 | Mechanism used to implement the random allocation sequence |
| Implementation | 9 | Page 5 | Who generated the allocation sequence, who enrolled participants, who assigned participants to interventions |
| Blinding | 10a | Page 5 | Whether who was blinded after allocation assignments |
|  | 10b | Page 5 | If blinded, description of how |
| **Statistical Methods** |  |  |  |
| Statistical methods | 11a | Page 8 | Statistical methods used to compare groups for primary and secondary outcomes |
|  | 11b | Page 8 | Methods for additional analyses |
| **Results** |  |  |  |
| Participant flow | 13a | Page 9, Figure 3 | Flow of participants through each stage |
|  | 13b | Page 9, Table 1 | Baseline characteristics for each group |
| Recruitment | 14a | Page 9 | Dates defining the periods of recruitment and follow-up |
|  | 14b | Page 9 | Why the trial ended or was stopped |
| Numbers analysed | 15 | Page 9 | Number of participants in each group included in primary analysis |
| Outcomes and estimation | 16 | Page 9-11 | For each primary and secondary outcome, results for each group |
| Ancillary analyses | 17 | Page 11-12 | Results of any other analyses performed |
| Harms | 18 | N/A | All important harms or unintended effects in each group |
| **Discussion** |  |  |  |
| Limitations | 19 | Page 12-13 | Trial limitations, addressing sources of potential bias |
| Generalizability | 20 | Page 13 | Generalizability (external validity) of the trial findings |
| Interpretation | 21 | Page 13-14 | Interpretation consistent with results, balancing benefits and harms |
| **Other Information** |  |  |  |
| Registration | 22 | Title Page | Registration number and name of trial registry |
| Protocol | 23 | N/A | Where full trial protocol can be accessed |
| Funding | 24 | Page 14 | Sources of funding and other support |
