# Supplementary material for: Advancing anesthesiology trainee proficiency in airway management via simulation-based training: a non-hypoxic apnea duration approach
Source: PeerJ. 2025 Jun 4;13:e19555. doi: 10.7717/peerj.19555 (PMC12145085; doi:10.7717/peerj.19555)
Supplement: Supplemental Information 3 [file peerj-13-19555-s003.docx]

**Clinical Trial Protocol**

**Study Title**

Advancing Anesthesiology Trainee Proficiency in Airway Management via Simulation-Based Training: A Non-hypoxic Apnea Duration Approach

**Registration Details**

- Registry: Chinese Clinical Trial Registration Center
- Registration Number: ChiCTR2200065877
- Registration Date: 17/11/2022

**Study Objectives**

**Primary Objective**

To evaluate the impact of incorporating non-hypoxic apnea duration awareness in simulation-based airway management education for anesthesiology trainees.

**Secondary Objectives**

1. Assess changes in clinical competence through modified DOPS scores
2. Measure trainee satisfaction with the simulation training approach
3. Evaluate performance improvements in specific airway management domains

**Study Design**

- Type: Quasi-experimental study
- Allocation: Randomized 1:1 allocation
- Blinding: Independent evaluator blinded to group assignment

**Participant Selection**

**Inclusion Criteria**

- Anesthesiology undergraduate students
- Age: 18-25 years
- Completed two-month theoretical and bedside internship course

**Exclusion Criteria**

- Less than three months of internship experience
- Previous tracheal intubation experience
- Withdrawal during study period
- Failure to complete simulation within specified timeframe

**Intervention**

**Intervention Group**

- Receive simulation with non-hypoxic apnea duration information
- Real-time countdown of 247 seconds until SpO2 reaches 90%
- Standardized difficult airway scenario

**Control Group**

- Conventional simulation training
- No specific non-hypoxic apnea duration information provided

**Outcome Measures**

**Primary Outcome**

- Modified Direct Observation of Procedural Skills (DOPS) assessment
- Evaluated across 10 distinct performance domains

**Secondary Outcome**

- Satisfaction questionnaire
- 10 domains assessed using 5-point Likert scale

**Statistical Analysis Plan**

- Sample size calculation:
  - Effect size: 0.8
  - α error: 0.05
  - Power: 0.8
- Comparative statistical tests:
  - Independent sample t-test for normally distributed data
  - Non-parametric tests for non-normal distributions
  - Chi-square test for categorical variables
- Software: SPSS 22.0
- Significance level: P < 0.05

**Ethical Considerations**

- Approved by Ethics Committee of Shanghai General Hospital (No. 2022KY093)
- Written informed consent from all participants
- Confidentiality of participant data
- No physical risk to participants

**Timeline**

- Study Period: December 2022 to March 2023
- Participant Recruitment: Consecutive sampling
- Simulation Sessions: Standardized protocol
- Data Collection and Analysis: Immediate post-simulation assessment

**Limitations and Considerations**

- Single-center design
- Small sample size
- Short observation period
- Limited generalizability

**Dissemination Plan**

- Manuscript preparation
- Presentation at relevant medical education conferences
- Publication in peer-reviewed journal

**Funding and Conflicts of Interest**

- No external funding reported
- Authors declare no competing interests
